# Automatic detection of CO_2_ rebreathing during BiPAP ventilation

**DOI:** 10.1038/s41598-024-63609-4

**Published:** 2024-08-17

**Authors:** Zbigniew Szkulmowski, Dominique Robert, Joanna Karłowska-Pik, Laurent Argaud

**Affiliations:** 1 Department of Anesthesiology and Intensive Care, Antoni Jurasz University Hospital nr 1, Ul. Sklodowskiej Curie 9, 85-094 Bydgoszcz, Poland; 2 Medical Intensive Care, Pavillon N, Hospices Civils de Lyon, Groupement Hospitalier Edouard Herriot, Lyon-Nord Medical School, University Claude Bernard Lyon I, 5 Place d’Arsonval, 69003 Lyon, France; 3 Faculty of Mathematics and Computer Science, Nicolaus Copernicus University in Toruń, Ul. Chopina 12/18, 87-100 Toruń, Poland

**Keywords:** BIPAP ventilation, CO_2_ movement, CO_2_ rebreathing, Continuous CO_2_ rebreathing detection, Artificial neural networks, Physiology, Biological techniques, Bioinformatics, Therapeutics

## Abstract

Carbon dioxide rebreathing (CO_2_ rebreathing) significantly influences respiratory drive and the work of breathing during BiPAP ventilation. We analyzed CO_2_ movement during BiPAP ventilation to find a method of real time detection of CO_2_ rebreathing without the need of CO_2_ concentration measurement sampled from the circuit (method expensive and not routinely used). Observational study during routine care in 15 bed university hospital ICU. At 18 patients who required BiPAP ventilation, intubated or during noninvasive ventilation, during weaning period airflow, pressure and CO_2_ concentration signals were registered on both sides of venting port and 17 respiratory parameters were measured or calculated for each of 4747 respiratory cycles analyzed. Based on CO_2_ movement (expiration–inspiration sequences) 3 types of cycle were identified, type I and II do not induce rebreathing but type III does. To test differences between the 3 types ANOVA, t-tests, and canonical discriminant analysis (CDA) were used. Then a multilayer perceptron (MLP) network, a type of artificial neural network, using the above parameters (excluding CO_2_ concentration) was applied to automatically identify the three types of respiratory cycles. Of the 4747 respiratory cycles, 1849 were type I, 1545 type II, and 1353 type III. ANOVA and t-tests showed significant differences between the types of respiratory cycles. CDA confirmed a correct apportionment of 93.9% of the cycles; notably, of 97.9% of type III. MLP automatically classified the respiratory cycles into the three types with 98.8% accuracy. Three types of respiratory cycles could be distinguished based on CO_2_ movement during BiPAP ventilation. Artificial neural networks can be used to automatically detect respiratory cycle type III, the only inducing CO_2_ rebreathing.

## Introduction

Since their introduction in the 1990s, Bilevel Positive Airway Pressure (BiPAP) devices have been widely used for mechanical ventilation in both hospital and home settings^1–3^. One of the benefits of using BIPAP ventilation is the ability to use single-limb systems with a passive leak port, which are simpler, lighter, more comfortable for the patient and less expensive than classic double-limb or single-limb systems with an external expiratory valve. Unfortunately, the use of single limb systems with a leak port is associated with an increased risk of CO_2_ rebreathing when BiPAP is used^4,5^.

Numerous factors can contribute to CO_2_ rebreathing, including low EPAP, intubation, low VT, high frequency of ventilation and position of the exhalation port^6–8^.

The clinical consequences are not clear but are considered negative in the context of hypercapnic respiratory failure as these factors increase PaCO_2_ levels and respiratory drive and consequently the work of breathing, which may explain the deterioration of ventilator-patient synchrony, specially important during noninvasive ventilation and, as a result, clinical noninvasive ventilation failure^5^.

Currently, the only way to detect and to confirm CO_2_ rebreathing is to continuously measure the inspiratory CO_2_ concentration sampled from the circuit close to the intentional leak port on the patient side. This is expensive and complicated and is not routinely used. At the same time, there are several parameters of mechanical ventilation related to pressure, flow and volume that are routinely and continuously registered by modern ventilators that could influence CO_2_ rebreathing for each breath.

It therefore seems likely that the analysis of some of these parameters could help detect CO_2_ rebreathing without having to measure CO_2_.

As a consequence, it seems possible in the future to modify the ventilator software in such a way that, without the need for hardware modifications, the ventilator will automatically detect and warn of the method of ventilation leading to CO_2_ rebreathing.

## Methods

This observational study was performed during routine patient care in an adult intensive care unit. Ethics approval was obtained from the local institutional review board, Comité de Protection des Personnes Sud-Est II in Lyon, France. Patients provided informed written consent for their participation, and the study was performed in accordance with the ethical standards of the 2013 World Medical Association Declaration of Helsinki^9^.

### Patients and ventilator settings

Records were performed on 18 patients stable and conscious who were intubated or under noninvasive ventilation using an inflatable air cushion (oronasal Tyco™ mask; Mallinckrodt DAR, Mirandola, Italy). The BiPAP ventilator (Vision^®^; Respironics Inc., Murrysville, PA, USA) was used in pressure support mode with a single limb circuit that comprised an exhalation port near the intubation tube or the mask (Whisper Swivel device; Respironics). The inspiratory positive airway pressures were chosen to deliver a tidal volume (V_T_) of 8–10 ml/kg. For patients with no intrinsic positive end-expiratory pressure (PEEPi), 4 cm H_2_O expiratory positive airway pressure (EPAP) was used, which is the lowest possible with that ventilator. If PEEPi was present and detected on the ventilator screen, the EPAP was adjusted to obtain zero end-expiratory flow without exceeding 9 cmH_2_O. The fraction of inspired oxygen (FiO_2_) was adjusted to maintain a minimal SpO_2_ of 93%.

### Recordings

**Figure 1:**
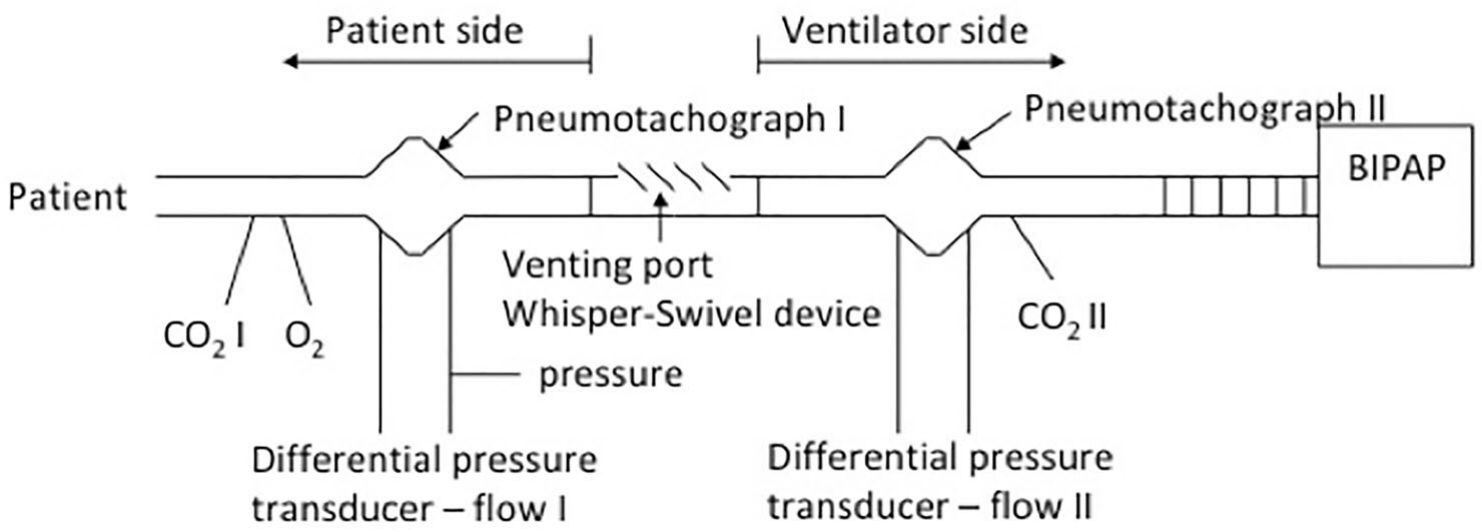
Measurement line. Measuring sensors placed on both sides of the leak port (Whisper–Swivel device). Patient side—part of the ventilator circuit between the leak port and the patient, Ventilator side—part of the ventilator circuit between the leak port and the ventilator, BIPAP—ventilator BIPAP. Measurements after Patient side: Pneumotachograph I—flow measurement Flow I using a differential pressure transducer, on one arm of the transducer a measurement of the pressure in the respiratory tract is performed after Patient side, CO_2_ I and O_2_—measurement of CO_2_ and O_2_ values. Measurements after Ventilator side: Pneumotachograph II—measurement of Flow II flow using a differential pressure transducer, CO_2_ II—measurement of CO_2_ values after Ventilator side.

Recordings that lasted from 15 to 30 min were obtained from each patient using the Biopac^®^ MP100 apparatus (Biopac Systems Inc., Santa Barbara, CA, USA). Calibrated pressure, airflow, O_2_, and CO_2_ (aspirating capnograph device) were sampled 2 cm away from both sides of the exhalation port (Fig. 1).

To measure CO_2_ concentration, the Biopac^®^ MP 100 measuring line was integrated with the CO_2_ measurement module (CO2100C) and the AFT20 kit for gas analysis recommended by the manufacturer. The AFT20 delay is 2.4 s, based on 1.5 mm ID tubing at 1.8 m and assuming 100 ml/min (factory preset) module gas sampling flow rate. The internal volume of the gas sampling interface kit is about 4.0 ml (PCV sample line 3.336 ml, NAFTION^®^ dryer 0.386 ml, misc. tubing/junctions 0.278 ml).

The manufacturer of the measuring line recommends using a suction speed (pump capacity) ranging from 50 ml/min to 200 m/min.

In our study, the air suction rate from the ventilatory circuit was increased to 150 ml/min, which shortened the delay time to 1.6 s according to the formula:

$$ \left( {{6}0\,{\text{s}}/{\text{min}}} \right) \, \times \, \left( {{4}\,{\text{ml}}} \right) \, / \, \left( {{15}0\,{\text{ml}}/{\text{min}}} \right) \, = { 1}.{6}\,{\text{s}} $$

According to these calculations, for the proper phasing of the pressure, air flow and CO_2_ concentration curves, the CO_2_ concentration curves: on the patient's side and on the ventilator's side were shifted by 1.6 s (withdrawn in the timeline) relative to the other curves.

Analyses of respiratory cycles and respiratory parameters were made breath by breath, after phasing CO_2_ curves with flow and pressure, using Biopac^®^ Acq-Knowledge^®^ 3.7 software. The beginning and end of each inspiration and expiration were defined at the points where flow crossed the zero line.

Respiratory cycles were analyzed during expiration and inspiration, on patient’s and ventilator side. For each analyzed respiratory cycle respiratory mechanics parameters were measured or calculated for the patient’s and ventilator side. For the patient’s side these were: inspiratory maximal pressure (insp maximal pressure Pat side), Positive end expiratory pressure, inspiratory time (Insp time), expiratory time (Exp time), frequency of breathing per minute, integral of surface under a pressure curve during inspiration (insp surface under pressure curve Pat side) and inspiratory (insp tidal volume Pat side) and expiratory (exp tidal volume Pat side) tidal volume as an integral under the flow curve, inspiratory (insp maximal flow Pat side) and expiratory (exp maximal flow Pat side) maximal flow. For the ventilator side: inspiratory (Insp tidal volume Vent side) and expiratory (Exp tidal volume Vent side) tidal volume, inspiratory (Inspiratory maximal flow Vent side) and expiratory (Expiratory maximal flow Vent side) maximal flow. The volume of CO_2_ inhaled at each breath (Insp CO_2_ volume per breath) was calculated for patient’s side by integrating the CO_2_ flow curve, defined as the product of inspiratory airflow and CO_2_ concentration curve.

After initial visual analysis of several recordings using Acq-Knowledge 3.7 software reflecting the CO_2_ movement (mainly gas flow and CO_2_ concentration curves on both sides of the leak port) in the respiratory circuit in it’s patient’s and ventilator side we have made some statements necessary for the further analysis.

### Classification of the three types of breath cycles

Ventilation, regardless of its type, is inevitably associated with CO_2_ rebreathing, which we can call physiological. During spontaneous ventilation it is the CO_2_ inhalation contained in the anatomical dead space, increased by the apparatus dead space during mechanical ventilation. At the leak circuit it is a level to leak port, including the patient part of the circuit (Fig. 1).

The CO_2_ rebreathing during BIPAP ventilation is a phenomenon of inhaling an extra CO_2_ from the ventilator part of the circuit during inspiration.

Amount of CO_2_ in inspired, atmospheric air is negligible; thus, CO_2_ rebreathing during inspiration should depend only on the CO_2_ exhaled during the previous expiration. For the purposes of this study, a breathing cycle was considered and analyzed as expiration-inspiration sequences. Only the CO_2_ from expiratory air exhaled into the ventilator side of the circuit, still present there at the end of expiration and re-inhaled during inspiration was considered. Each breathing cycle was analyzed visually for the presence of CO_2_ on the ventilator side of the leak port during expiration and on the patient side during inspiration.

When assessing which class a given respiratory cycle belongs to, both methods were used, visual and classical, i.e. the assessment of the level of CO_2_ taken from the breathing circuit.

**Figure 2:**
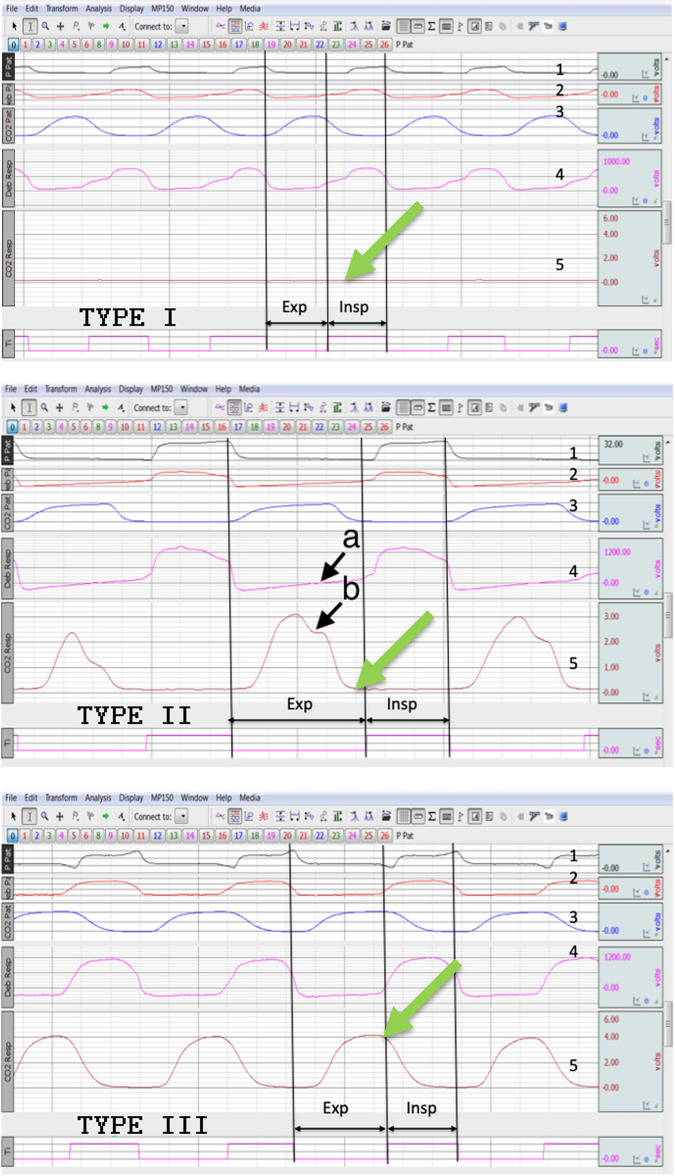
3 types of respiratory cycles: The curves show: 1—pressure, patient side, 2—flow, patient side, 3—patient side, CO_2_ concentration, 4—flow, ventilator side, and 5—ventilator side, CO_2_ concentration. *Exp* expiration, *Insp* inhalation. Vertical lines mark the beginning and end of expiration and inspiration in the breathing cycle, crossing the point of 0 flow on the patient-side flow curve (curve 2). The height of the individual curves has been manually modified for better visualization and does not correspond to their actual values. Black arrows with "a" and "b" markings on the curves for the type II breathing cycle: 4—flow, ventilator side and 5—CO_2_ concentration, ventilator side, mark the moment of reversal of the flow direction during exhalation—from the initial expiratory flow from the patient in towards the flow of the ventilator, in the second phase of exhalation, towards the patient and the Whisper–Sviwel device. The reversal of the direction of expiratory flow in type II results from the decreasing values of the patient's expiratory flow, which at indicated moments becomes smaller than the constant flow towards the patient generated by the BIPAP ventilator, necessary to maintain a constant expiratory positive airway pressure EPAP. The green arrows indicate the CO_2_ concentration values on curve 5 at the start of the next inspiration: for type I and type II these values are 0, while they are positive for type III. The illustrations are print screens of actual measurements performed on patients using AcqKnowledge 3.7 (Biopac System Inc., Santa Barbara, CA, USA).

The classical evaluation is based on the evaluation of the CO_2_ concentration in the respiratory circuit on both sides of the Whisper-Sviwel device (Fig. 1). The reflection of CO_2_ concentration are 2 curves: the first—CO_2_ concentration on the patient side and the second—CO_2_ concentration on the ventilator side. To assess whether the cycle belongs to particular classes, we followed the second CO_2_ curve. The distinction between different types of breathing cycles is presented on Fig. 2 and relies on:

Type I—when CO_2_ does not appear during exhalation on the ventilator side, the CO_2_ curve on the ventilator side is flat and takes the values of 0 (green arrow, curve 5—Fig. 2).

Type II—when CO_2_ appears during exhalation on the ventilator side, but due to the constant flow from the ventilator towards the patient (to maintain a constant end expiratory pressure EPAP), it is completely flushed out through the exhalation port before the next inspiration. Positive values appear on the CO_2_ curve, but they drop to 0 before starting the next inspiration (green arrow on curve 5, Fig. 2). In this type, the expiratory flow is two-phase, during expiration the direction of the airflow is reversed (curve 4, Fig. 2). Arrow "a" shows the moment of flow reversal (curve 4, Fig. 2). This moment is also visible on the ventilator's CO_2_ curve—refraction in curve 5 (arrow "b", Fig. 2).

Type III—when CO_2_ appears during exhalation on the side of the ventilator, but is not completely flushed out during exhalation. At the start of the next inspiration, exhaled CO_2_ remains at the ventilator part of the circuit and is inhaled by the patient during next inspiration. The CO_2_ curve at the start of the next inspiration is still positive (green arrow in curve 5, Fig. 2). Only in type III of breathing cycle CO_2_ rebreathing appears.

The evaluation of the cycles included in the analysis was carried out independently by 3 authors. During the qualification of the cycles for analysis, the possibility of patient identification was disabled. The analysis eliminated the cycles with deteriorated registration quality, significant disturbances of the respiratory rhythm (e.g., patient's agitation or movements), difficult to assess due to significantly increased air leakage (episodes of increased leakage in non-invasive ventilated patients) or airway procedures (e.g. suctioning in invasively ventilated patients). "Borderline" respiratory cycles were also eliminated, that is, which cannot be indisputably classified into one of the 3 types.

In this way, in the process of qualifying the respiratory cycles for analysis, all cycles recorded in 2 patients were eliminated, so finally the respiratory cycles from 16 patients were analyzed.

### Identification, analysis and automatic recognition of the three types of respiratory cycle

Each breath cycle was visually analyzed and categorized as type I, II or III. Then, 2 tests were performed to compare the three types of breath cycles. First, ANOVA compared the 17 parameters in the three types of cycles; second, if significant differences were found by ANOVA, an unpaired Student’s t-test was used to compare pairs of cycle types (Matlab Software, MathWorks^®^, MA, USA). Differences were considered significant for p < 0.05. Second a canonical discriminant analysis (CDA) was performed to appreciate correct categorization of the cycles into the three respiratory cycle types.

To test whether the three types of respiratory cycles could be identified automatically, we used a multilayer perceptron (MLP) network, which is a type of artificial neural network (PS IMAGO PRO 8 software based on the analytical engine IBM SPSS Statistics v. 28).

The totality of the classified breath was split randomly in 10 samples of equal size to conduct tenfold cross-validation procedure. Then 10 MLP models were built. Each time one of 10 samples (sequentially) was treated as a test set and the remaining 9 samples were together treated as a training set.

Each breath is characterized by the 16 variables tested by ANOVA (but excluding the CO_2_ concentration variable) which were predictors for the models. These variables were standardized. The target variable was the type of respiratory cycles.

The MLP models had two hidden layers with sigmoid function as the activation function. The number of neurons in these layers was chosen automatically. Because the target variable is categorical, for the output layer the softmax function was used as the activation function. From every training sample 70% of observations were used to correct weights associated with the connections between neurons in the MLP model and the remaining 30% of observations were used to define the stopping condition, what is a standard procedure for MLP model in IBM SPSS Statistics software.

Each of 10 models was tested on the corresponding test set and each time the accuracy of the model and the sensitivity as well as precision for each type of respiratory cycle were calculated. At the end they were averaged. For each type of respiratory cycle also the ROC curve was drawn and the area under the curve was calculated. Here the pooling strategy for ROC performance for cross-validation was used^10^.

### Ethics approval and consent to participate

This observational study was performed during routine patient care in an adult intensive care unit. Ethics approval was obtained from the local institutional review board. Patients provided informed written consent for their participation, and the study was performed in accordance with the ethical standards of the 1964 Declaration of Helsinki.

Trial registration: The study was not registered as it does not meet the ICTRP criteria of a clinical study: it is an observational study, only data from the respiratory system of the ventilator during routine treatment activities were recorded, without any interventions in relation to the patient and treatment.

## Results

**Figure 3:**
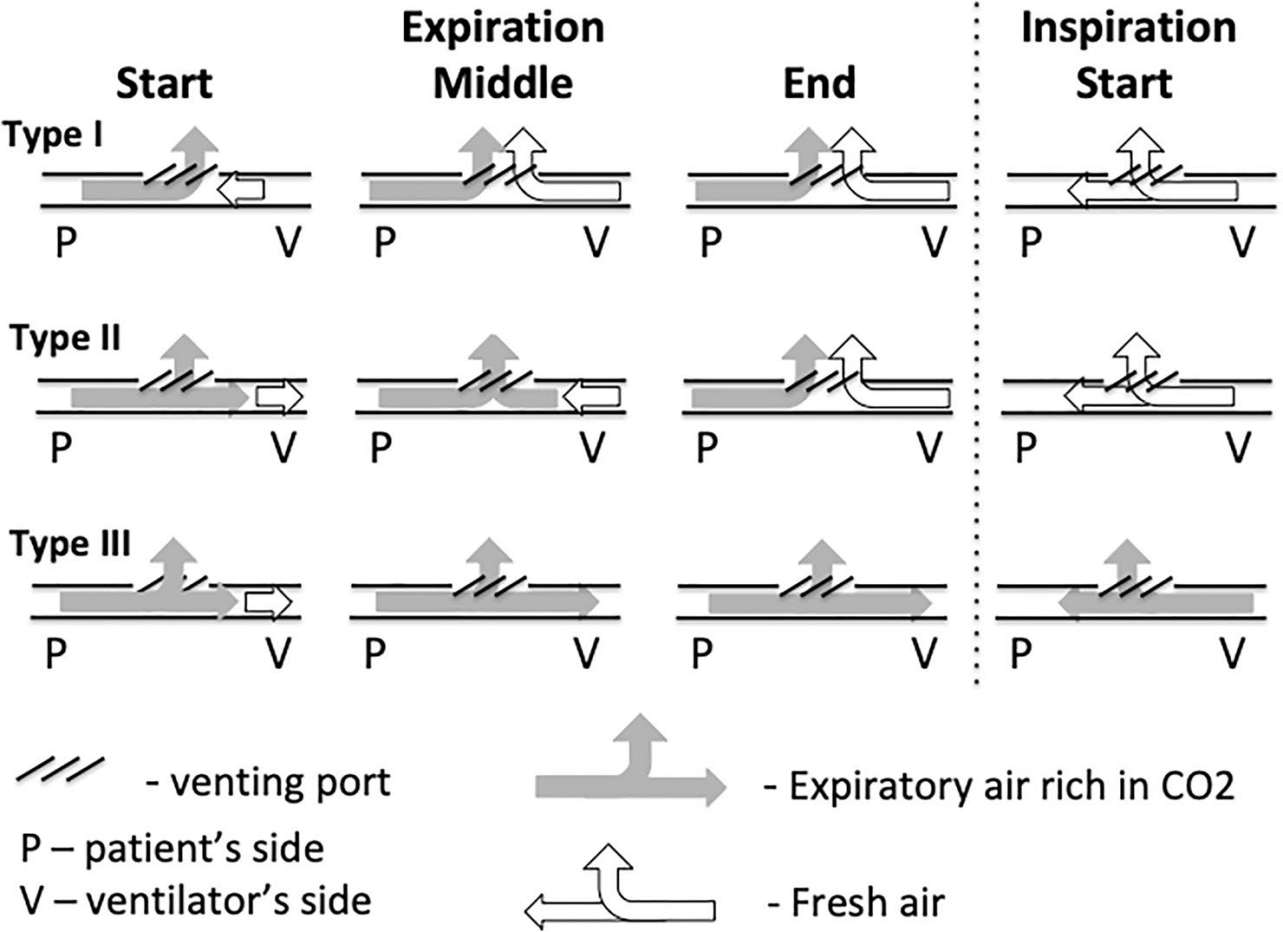
A model of CO2 movement for the three types of respiratory cycles during BiPAP ventilation. Type I—when CO_2_ does not appear during exhalation on the ventilator side, all exhalation air is removed during exhalation through the leak port, CO_2_ does not appear on the patient's side during exhalation, Type II—the exhalation is so intense that CO_2_-rich exhalation air appears during exhalation on the ventilator side, but due to the constant flow from the ventilator towards the patient (to maintain a constant end expiratory pressure EPAP through the BIPAP device), it is completely flushed out through the exhalation port before the next inspiration. Type III—here also CO_2_ appears during exhalation on the side of the ventilator, but is not completely flushed out during exhalation. At the start of the next inspiration, exhaled CO_2_ remains at the ventilator part of the circuit and is inhaled by the patient during next inspiration.

Based on the analysis of pressure, flow and CO_2_ concentration curves on the patient and ventilator side, a model of CO_2_ movement in the leak circuit during BIPAP ventilation was created. It is presented in Fig. 3.

Eighteen patients were studied. Thirteen of them were male. Mean age was 54 ± 19 years, the mean time of mechanical ventilation before the study was 8.2 ± 6.6 days. The table with the characteristics of individual patients has been included in the Supplementary Materials. 11,821 respiratory cycles were manually and visually analyzed. Out of them 4747 were qualified to one of 3 types as the best fitting the model of each type: 1849 of them were qualified as type I (38.9%), 1545 as type II (32.5%) and 1353 as type III (28.5%). 3224 (67.9%) respiratory cycles were registered at intubated patients, 1523 (32.1%) during noninvasive ventilation.

The number of respiratory cycles included in the analysis for individual patients ranged from 588 to 29 (mean 296.7 ± 288.5 SD, median 288.6).

Table 1 shows the 17 parameters that were measured or calculated for every breath cycle and compares them in the three categories.

**Table 1:** Values (means ± SDs) of the studied respiratory parameters. ANOVA showed significant differences between the parameters in the three types of respiratory cycles (*p* < 0.0001 for each parameter). Stepwise multiple comparison (Neuman–Keuls test) for differences between types: I, II and III: ^a^*p* < 0.05 type II vs type I, ^b^*p* < 0.05 type III vs type I, ^c^*p* < 0.05 type III vs type II. *Insp* inspiratory, *Exp* expiratory, *Pat* patient, *Vent* ventilator.

Respiratory parameter	All n = 4747	Type I n = 1849	Type II n = 1545	Type III n = 1353
Insp CO_2_ volume per breath, ml	1.7 ± 3.3	0.48 ± 0.25	0.39 ± 0.31	4.92 ± 4.88^b,c^
Insp maximal pressure Pat side (cm H_2_O)	22 ± 6.8	24 ± 5.4	26.3 ± 4.6^a^	14.4 ± 3.5^b,c^
.Insp time (s)	1.1 ± 0.6	1.0 ± 0.8	1.1 ± 0.4^a^	1.2 ± 0.5^b,c^
Exp time (s)	1.4 ± 0.6	1.2 ± 0.5	1.7 ± 0.7^a^	1.5 ± 0.6^b,c^
Insp surface under pressure curve Pat side (cm^2^)	17.3 ± 7.9	16.4 ± 7.9	22.6 ± 6.5^a^	12.6 ± 5.3^b,c^
Exp surface under pressure curve Pat side (cm^2^)	13.1 ± 6.3	12 ± 5	17.4 ± 7^a^	9.7 ± 3.9^b,c^
Insp tidal volume Pat side (ml)	519 ± 261	394 ± 238	590 ± 200^a^	610 ± 283^b,c^
Exp tidal volume Pat side (ml)	442 ± 238	252 ± 87	537 ± 165^a^	594 ± 270^b,c^
Insp tidal volume Vent side (ml)	986 ± 445	825 ± 472	1141 ± 347^a^	1027 ± 435^b,c^
Exp tidal volume Vent side (ml)	87.3 ± 198	− 69 ± 83.6	81.5 ± 94.7^a^	307 ± 191^b,c^
Insp maximal flow Pat side (ml/s)	726 ± 174	683 ± 223	799 ± 114^a^	703 ± 116^b,c^
Expiratory maximal flow pat side (ml/s)	515 ± 143	385 ± 106	617 ± 99^a^	575 ± 81^b,c^
Inspiratory maximal flow Vent side (ml/s)	1243 ± 249	1215 ± 296	1394 ± 171^a^	1108 ± 137^b,c^
Expiratory maximal flow Vent side (ml/s)	263 ± 151	106 ± 76	341 ± 91^a^	387 ± 84^b,c^
Frequency of breathing per minute/min	27 ± 8.2	31 ± 8.2	24 ± 8^a^	25 ± 6.1^b,c^
Positive end expiratory pressure, cm H_2_O	9.2 ± 2	10.1 ± 1.4	10.4 ± 1.3^a^	6.6 ± 0.3^b,c^
Invasive/Non-Invasive	3324/1523	1196/653	1026/519	1002/351

**Figure 4:**
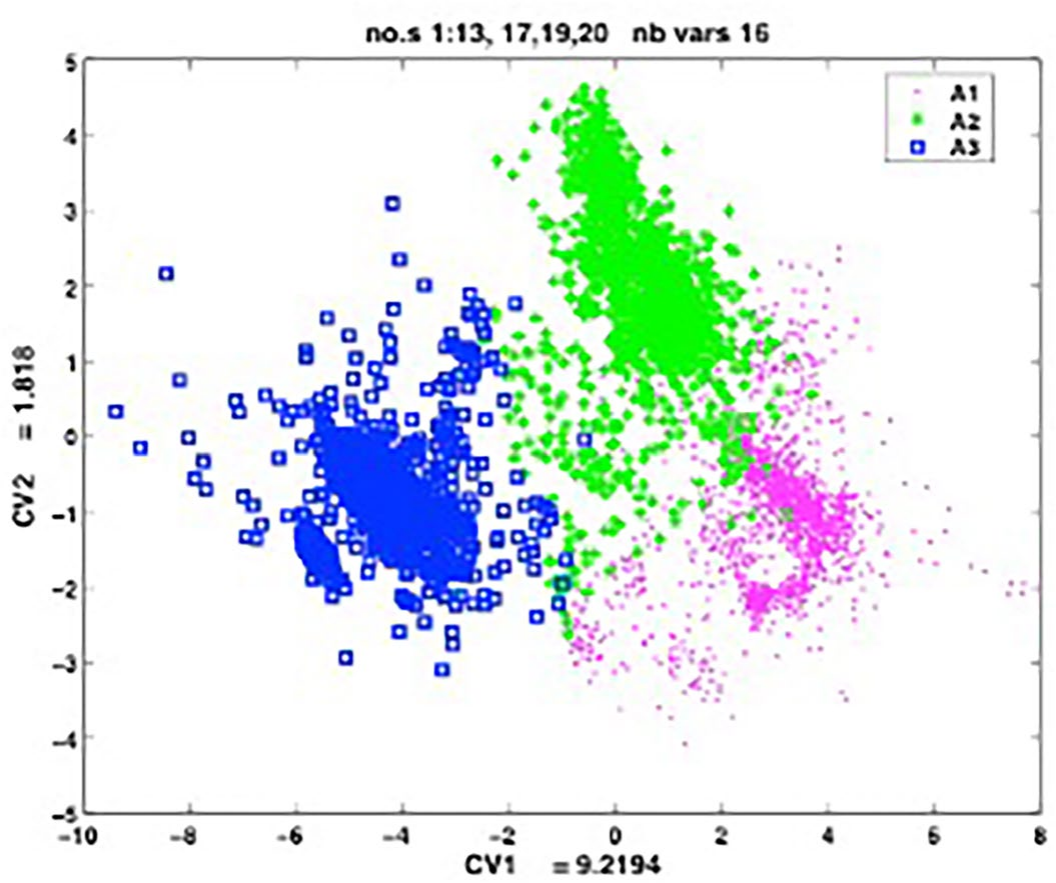
Discriminant analysis for 16 measurements studied. The CDA method computes for our 16-dimensional data vectors their two-dimensional representation (CD1, CD2) which may be displayed in the plane < CD1, CD2 > . The representation obtained for our n = 4747 data vectors is shown. Fishers index for CV1 equals 9.2194 and Lambda Wilk’s: 0.0347 which means a good discriminative power. Points A1, A2 and A3 refer to respiratory cycles type I, II and III, respectively.

ANOVA showed that all parameters differed significantly among the three types; in addition, pairwise comparisons also showed significant differences for all parameters for different breath types except for inspiratory CO_2_ between type I and II breaths. CDA was performed using the same variables and showed that 93.9% of the breaths according to breath type were correctly categorized (Fig. 4).

Notably, CDA correctly identified 97.9% of type III breaths, which is important because type III breaths involve CO_2_ rebreathing (Table 2).

**Table 2:** Classification matrix using canonical discriminant analysis (CDA) to analyze 16 parameters. The first column shows the true number of breaths in each group (type I, II, and III). Columns 3, 4, and 5 show how CDA categorized the breaths to each group.

No of breaths in each group	% of breaths that were correctly classified	Breaths categorized by CDA		
		Type I	Type II	Type III
I 1849	94.32	1744	0	105
II 1545	90.1	117	1392	36
III 1353	97.86	29	0	1324
All 4747	93.95	1890	1392	1465

The MLP network trained and tested 10 times in a cross-validation procedure showed 98.8% accuracy for categorizing the cycles type with a sensitivity 99.0%, 97.9%, and 99.6% and precision 98.6%, 98.4%, and 99.5% for types 1, 2, and 3, respectively (Table 3).

**Table 3:** MLP model quality obtained as a result of tenfold cross-validation procedure.

Measure	Min	Mean	Max
Accuracy	97.5%	98.8%	99.4%
Sensitivity for type 1	97.1%	99.0%	100.0%
Sensitivity for type 2	95.8%	97.9%	98.8%
Sensitivity for type 3	98.5%	99.6%	100.0%
Precision for type 1	97.3%	98.6%	100.0%
Precision for type 2	96.5%	98.4%	99.4%
Precision for type 3	98.4%	99.5%	100.0%

The AUC for each cycle type were as follows: 0.999, 0.998, and 1.000, respectively (Table 4).

**Table 4:** Area under curve (AUC) for MLP model obtained as a result of tenfold cross-validation procedure.

Type of respiratory cycle	AUC	95% confidence interval for AUC
1	0.999	0.999–1.000
2	0.998	0.997–0.999
3	1.000	0.999–1.000

## Discussion

This study analyzed the characteristics of 4747 breaths recorded on patients who were ventilated with a single limb circuit with a leak port. First, we categorized the breaths into three types based on the CO_2_ motion. Second, we demonstrated that simple characteristics that can be measured on pressure and flow curves differ according to the three types of CO_2_ motion. Third, we showed that we could use a artificial neural network technique, multilayer perceptron (MLP) network, to recognize each type of breath. The results suggest that by analyzing and calculating parameters routinely measured by modern ventilators, it will be possible, after appropriate refinement of the analysis algorithm, to monitor the type of each breath, which allows for adjustment of the ventilator settings to minimize the risk of re-inhalation of CO_2_. With the computing capabilities of modern ventilators, it should be easily calculated in real time to warn the medical staff of the increased risk of CO_2_ rebreathing.

Importantly, this study considered the complexity of the actual continuous interactions between the effects of mechanical ventilation and their effects on the physiological regulation of ventilation in terms of drive, frequency, and the inspiratory/expiratory ratio and the possibility of non-intentional leaks. It is important to emphasize how the calculations were performed. At the beginning, we made one very important assumption. As the amount of inhaled CO_2_ in ambient air is negligible, during mechanical ventilation using a sealed respirator system, CO_2_ rebreathing during inspiration should depend mainly on the amount of CO_2_ exhaled during the previous exhalation. Only the CO_2_ from expiratory air exhaled into the ventilator side of the circuit, still present there at the end of expiration and re-inhaled during inspiration was considered as CO_2_ rebreathing. Therefore, for the purposes of this study, a breathing cycle was considered and analyzed as expiration–inspiration sequences. Then, each breath type was identified “visually” according the CO_2_ motions described at Fig. 2. Then we found that the main characteristics showed significant differences according to the type of breath, as confirmed by ANOVA and CDA, allowing us to correctly differentiate each type (with 97.9% accuracy for type III breaths). Finally, we used an artificial neural network method to analyze and categorize the three types of breath.

Our sample comprised patients who were intubated or who were non-invasively ventilated via a mask that covered the mouth and nose. The distribution of breath types in the intubation and noninvasive ventilation groups, respectively, were as follows: type I, 64% and 36%; type II, 66% and 34%; and type III, 74% and 26%. It was possible that the process needed to identify the breath type might differ according to intubation versus mask ventilation status; however, since the identification was close to 100% accurate using the MLP network, this does not appear to be a concern. Indeed, this method seems to be usable regardless of whether the patient is intubated or is using an oronasal mask.

The definition of CO_2_ rebreathing adopted in our work and placed in the Method section requires a broader clarification—"The CO_2_ rebreathing during BIPAP ventilation is a phenomenon of inhaling an extra CO_2_ from the ventilator part of the circuit during inspiration". We adopted this definition because it corresponds to the specificity of mechanical ventilation in the BIPAP mode using a leak ventilatory circuit.

CO_2_ rebreathing is inherent in every type of ventilation, both spontaneous and mechanical ventilation.

During spontaneous breathing, CO_2_ rebreathing affects the entire anatomical dead space—during each subsequent inspiration, CO_2_ is inhaled again from the exhalation air contained in the upper respiratory tract at the end of exhalation.

In the case of mechanical ventilation, we must add an apparatus dead space to the anatomical dead space, which is where additional CO_2_ inhaled during each subsequent inspiration comes from.

For the purposes of our work, we adopted a definition in which we specify that the analyzed rebreathing CO_2_ will be additional CO_2_, in addition to that from the anatomical and apparatus dead space, which will be inhaled by the patient during inspiration. We did this for 2 reasons.

Firstly, equipment dead space is an inevitable phenomenon during mechanical ventilation. Manufacturers of medical equipment, including masks and ventilator tube systems, make efforts to minimize the size of this space, but its complete elimination is impossible for technical reasons. The size of the device dead space varies and depends on the type of equipment selected for a specific patient (including the size of the mask, the location of the exhalation port, …) but for patients ventilated with identical equipment it will be practically the same. And this assumption is correct regardless of the ventilation mode. However, only during mechanical ventilation conducted in the BIPAP mode in a valveless system using a leak port, we are dealing with additional CO_2_ appearing in the ventilatory circuit in the part distal to the leak port, as we showed in our work, only in type III respiratory cycles. So this is the phenomenon we analyze in our work. Secondly, we would like to emphasize that the phenomenon of CO_2_ rebreathing defined in this way during BIPAP ventilation does not depend on the size of the dead space apparatus. Equipment manufacturers try to reduce the size of the equipment's dead space by moving the leak port as much as possible towards the patient. In the newest masks, it is located on the mask itself. However, this does not change the essence of CO_2_ rebreathing because, as our work shows, including the diagram describing the movement of CO_2_ in the ventilatory circuit, it is caused by the passage of exhaust air from CO_2_ to the ventilator part of the circuit between the leak port and the ventilator and the impossibility of its complete removal. elimination through the leak port until the next inspiration begins. Therefore, this is a phenomenon typical of BIPAP ventilation and which occurs outside the equipment dead space, and for this reason our definition of CO_2_ rebreathing seems justified to us.

Another possible concern is that the type of breath could always be the same for a particular patient using specific ventilator settings, making continuous analysis not very relevant. However, we found that for each patient, the average percentage of breaths that showed significant rebreathing (FICO_2_ > 0.1%) was 45% ± 31%. This clearly indicates that each patient shows both rebreathing and non-rebreathing. This is illustrated on the patients’ records, some of which show the three types of breath during the same minute. In a previous study^8^, logistic regression analysis showed that rebreathing may depend both on the previous value of the end tidal level of CO_2_ (OR = 3.09) and on the instantaneous respiratory rate expressed as breaths per minute (OR = 1.19).

In that study, a leak valve was mandatory with the single limb circuit, which was the classical Respironics Whisper Swivel that is located on the circuit close to the intubation tube or to the mask. Some studies have shown that if there is a noninvasive interface, a leak incorporated into the mask itself decreases rebreathing in bench studies^6,7^. Of course, this makes breathing more efficient by decreasing the dead space of the mask and thus the VD/VT ratio. Nevertheless, the mechanism and the risk of rebreathing we describe here remain the same. In other words, this means that the amount of CO_2_ rebreathing only partially depends on the location of the leak port, whether on the mask itself or at some distance from it. To a greater extent, CO_2_ rebreathing depends on how much CO_2_-rich air will be exhaled to the circuit limb between the leak port and the ventilator (ventilator part) during exhalation, and the ability to remove CO_2_ from the system until the next inhalation, which depends on amount of CO_2_ in the system and expiration time.

Some studies propose that a plateau exhalation device or a non-rebreather valve be used to limit the risk of rebreathing and show that these kinds of devices can eliminate rebreathing at the expense of an increase in work of breathing and of expiratory resistance^11^. On the other hand, in patients with COPD, rebreathing increases during exercise; this is related to the high expiratory flow, which is not totally eliminated during shortened expiratory duration, which may increase in the respiratory frequency^12^. Notably, non-rebreather valves are not currently recommended, especially since a clinical trial showed negative results^11^.

One limitation of our study was that the method we described here allows only a qualitative measure of whether there is rebreathing; it does not actually quantify rebreathing. The obtained data potentially enable the use of quantitative analysis of the amount of CO_2_ rebreathing. It was not carried out because the authors believe that the purpose of the current work is to demonstrate the possibility of detecting CO_2_ rebreathing, and qualitative and quantitative analysis of CO_2_ rebreathing, in order to obtain reliable conclusions, will require the recruitment of a larger number of patients, selected for various diseases and their advancement, and it will be subject of further research.

The limitation of the study seems to be the manual assessment of individual respiratory cycles and assigning them to one of three types. As described in the Method section, this process, in order to minimize the risk of error, was performed independently by three authors who were also experienced clinical practitioners and researchers. It was based primarily on the analysis of recorded curves and the presence of CO_2_ on the ventilator side during exhalation. Type I included cycles during which no CO_2_ appeared at all on the CO_2_ curve on the ventilator side during exhalation, type II included cycles during which CO_2_ appeared on this curve but disappeared before the next inspiration began, and type III included cycles during which CO_2_ was present on the side of the ventilator not only during exhalation, but its presence also persisted at the beginning of the next inspiration and could therefore move towards the patient as CO_2_ rebreathing. This is illustrated in Fig. 3.

This "manual" way of analyzing respiratory cycles was necessary for several reasons. First of all, in our work we used a different method of analyzing respiratory cycles than the commonly used one. For the purposes of this work, we analyzed the respiratory cycle as an exhalation-inhalation sequence and not, as commonly assumed, inhalation-exhalation. This resulted from the logic of mechanical ventilation and CO_2_ movements in the leak circuit during BIPAP ventilation shown in Fig. 2. The presence of CO_2_ rebreathing and its magnitude in this mode of mechanical ventilation depends on the amount of CO_2_ entering the ventilator circuit behind the Whisper-Sviwel device (to the ventilator side) and the ability of the leak circuit to eliminate it from this part of the system until the next inspiration begins. Therefore, the lack of studies available in the literature using the method we used made it impossible to base our analysis on other or even automated methods of classifying respiratory cycles.

At the same time, it seems that the method of classifying respiratory cycles is transparent and the risk of misclassification is low. Additionally, this was one of the reasons why, among other things, to minimize this risk, we included only "ideal" cycles in the analysis, i.e. undoubtedly belonging to one of the 3 types.

The correctness of the method used to classify cycles into three types was confirmed in statistical tests.

Another element to consider is that only the "ideal" breathing cycles of each of the 3 types are selected for analysis. It was a deliberate procedure that allowed for a more complete determination of the differences between the different types of respiratory cycles. During real, clinical and bedside registrations, apart from "ideal" cycles, there will be many cycles with disturbed morphology, resulting from increased patient activity or leaks during non-invasive ventilation. It is likely that continuous analysis of all respiratory cycles, including "non-ideal" ones, would not yield as high a sensitivity as analyzing only "ideal" cycles. However, this does not change the clinical value of our work, as it indicates the possibility of developing algorithms, using the methodology and analytical techniques described in our article, detecting CO_2_ rebreathing also in non-ideal registrations.

The size of the study group may be a limitation of the work. The study included respiratory cycles obtained from the registration of only 18 patients. Additionally, according to the "Patient's characteristics" table in Supplementary Materials, this group was not homogeneous in terms of age, underlying disease or duration of mechanical ventilation. However, the authors do not consider this element to be a significant limitation, for several reasons.

The authors' main interest were respiratory cycles and their characteristics, described by several variables.

The aim of the study was to demonstrate that the assessment of only the parameters recorded by modern ventilators, i.e. the pressure and flow in the ventilator's respiratory system, may be sufficient to detect the risk of CO_2_ rebreathing without the need of additional CO_2_ measurement in the ventilator circuit.

For this purpose, the respiratory cycles were analyzed, which are never identical during assisted ventilation, such as ventilation in the BIPAP mode. Each respiratory cycle, also in an individual patient, is different from the others because its characteristics are influenced by many variables on the patient's side, such as: the force generated by the patient's respiratory muscles, the speed of inspiration, the use of expiratory muscles, the amount of secretions in the respiratory tract, the degree of arousal. psychomotor and many other factors. If a larger number of patients were examined and a larger number of respiratory cycles were analyzed, an even greater variety of cycles would probably be obtained than in the registered group of 4747 respiratory cycles analyzed. This does not change the fact that the recorded pool of approximately 4.5 thousand cycles turned out to be completely sufficient to obtain satisfactory results confirming the assumed thesis, i.e. the possibility of detecting type 3 breathing cycles without the need to measure CO_2_ in the ventilator circuit.

Explaining the mechanism of CO_2_ rebreathing formation and linking it with the underlying pathology would allow for a better setting of the ventilator's initial parameters in a specific patient, and perhaps in the future a step towards automating the process of adapting ventilation parameters to the patient's own breathing.

Such an analysis would require more registrations in a greater number of patients with different ventilation pathologies. Only in this case will we have a database of the size necessary to make the appropriate comparisons. Unfortunately, the current database of 18 patients is too small.

The choice of deep learning model requires explanation. In our current work, we wanted to show that the parameters we recorded are good predictors for predicting our target variable, which is the type of respiratory cycle, and a relatively simple MLP model is already sufficient to obtain good results of this prediction. It seems to me that it is always advisable to use an analytical tool that is the least complex and hardware-demanding (in terms of processor performance) so that the algorithm that analyzes rebreathing can be easily implemented on as many ventilators as possible.

Using a better model, as suggested, would probably give better results, but in the case of AUC it would be a correction of 0.001 (after all, our AUC is 0.998–1.000), so small and with no significant impact on the final results.

Another key point to keep in mind is the potential clinical importance of the amount of rebreathing that comes from the circuit and adds to the anatomical dead space ventilation that we identified. Some studies note that this is not a cause for concern, especially in noninvasive ventilation, due to non-intentional leaks^13^. Nevertheless, the impact of even a slight increase or decrease in PaCO_2_ and the impact of frequent and rapid changes in PaCO_2_ remain unknown. However, a change as small as 1 mmHg in the PaCO_2_ is a powerful stimulus in terms of regulating respiratory drive, and the PaCO_2_ level has important hemodynamic effects that are mediated mainly by sympathetic stimulation of pulmonary tension, peripheral tension, and cerebral blood flow^14–16^. On the other hand, clinical studies clearly show that in either acute or chronic hypercapnic respiratory failure, the aim of ventilator assistance is to decrease the PaCO_2_ level, which may be impeded if the BiPAP circuit induces rebreathing.

The choice of the type of capnometer used requires explanation. Determination of CO_2_ concentrations in exhaust air can be performed using mainstream or sidestream techniques.

Both techniques have their advantages and disadvantages. The mainstream technique is more accurate and the measurement is made at the point of gas flow, so the reading of the CO_2_ concentration value is immediate, in real time. The sidestream technique involves measuring the CO_2_ concentration in the air aspirated from the respiratory circuit using a thin tube into a capnograph where the measurement is performed. Therefore, the CO_2_ concentration reading is delayed by the time of gas flow from the ventilatory circuit to the capnograph and is a function of the internal volume of the supply tube and the flow rate of the pump aspirating air from the ventilatory circuit. In order to synchronize the CO_2_ concentration curve with the curves of other parameters measured in the ventilator circuit, i.e. pressure and flow, in the sidestream technique it is necessary to take this delay into account, i.e. shift the CO_2_ concentration curve (roll back on the time line) by the value of this delay.

The factors determining the use of the sidestream CO_2_ concentration measurement technique in our study are primarily the specificity of the mechanical ventilation performed in the studied patients. In some of the examined patients, ventilation was performed non-invasively, i.e. using a mask attached to the face. The condition for the effectiveness of such ventilation is to maintain the tightness of the connection between the mask and the patient's face. In the sidestream technique, the test requires the addition of a small and, above all, light plastic connector with a thin tube that discharges the tested gas to the analyzer located outside the ventilator tubing. This testing technique does not change the conditions for non-invasive ventilation.

If we use the mainstream technique, the CO_2_ concentration detector, much larger and heavier, should be placed directly on the ventilator tubing, between the mask and the venting port. The weight of the detector and the cable connecting to the analyzer would burden the mask and could result in loss of tightness of the ventilator-patient system and uncontrolled leaks disturbing the recording. The problem was important because we wanted the measurements in intubated and non-invasively ventilated patients to be comparable, so we maximized efforts to minimize leaks.

Moreover, in the mainstream technique it is only possible to analyze CO_2_ concentrations, while in our study we also analyzed O_2_ concentrations on the patient side. In the sidestream technique, unlike the mainstream technique, it is possible to analyze many gases, so this was one of the arguments for using this technique.

Of course, taking into account the measurement principle in the sidestream technique, differences in the maximum CO_2_ concentration value can also be expected due to the admixture of aspirated fresh air from the ventilator circuit. However, this phenomenon is observed primarily in the case of small tidal volumes, in recordings conducted in children. Our study was based on registrations conducted in adults with tidal volumes of several hundred ml/breath, so this phenomenon should not be significant.

An important issue is whether the values obtained by both techniques are comparable and whether different CO_2_ concentration values could be obtained using the mainstream technique instead of the sidestream technique.

There are few studies comparing the accuracy and comparability of both techniques during mechanical ventilation.

One of the few works is the analysis of Balogh et al.^17^, in which he showed that the results of the side and mainstream techniques do not differ significantly and are comparable when assessing end-tidal CO_2_ (PETCO_2_), intrapulmonary shunt fraction, ventilation-perfusion mismatch and volumetric CO_2_ values, i.e. those, which were assessed in our study.

Therefore, it seems that despite certain limitations of the sidestream measurement technique, the curve records obtained thanks to it and their accuracy are sufficient to confirm the theses assessed in our work.

Another problem is the impact of possible air leaks, which are inherent in non-invasive ventilation, on CO_2_ rebreathing. Some of our patients were non-invasively ventilated using a face mask.

Air leak during non-invasive ventilation obviously affects the amount of CO_2_ rebreathing.

This leak occurs between the mask and the patient's face, is always unidirectional and in the case of BIPAP ventilation causes two phenomena: (1) Removal of CO_2_-rich air from the equipment dead space and (2) Removal of CO_2_-rich air from the part of the ventilatory circuit distal to the leak port, which changes thus turning parts of cycles that could be type III cycles without this leak into type II cycles in which CO_2_ rebreathing will not occur.

From the point of view of CO_2_ rebreathing air leaks could be paradoxically beneficial phenomenon, but these benefits are obviously outweighed by unfavorable side effects in the form of respirator-patient desynchronization, reduced patient comfort or excessive drying of the inspiratory air.

Air leakage during BIPAP ventilation is more severe during inspiration, when the pressure in the ventilatory circuit is higher, but may also persist during exhalation.

This phenomenon also occurs in our study. As shown in Table 1, the Insp tidal volume Pat side is on average higher than the expiratory tidal volume Pat side, which is most likely due to leakage, although it was not noticeable. clinically.

However, it seems that the phenomenon of air leaks does not have a significant impact on the results obtained in our work.

This is mainly due to the method of analysis of respiratory cycles we used, i.e. in the adopted sequence of analysis in the order of exhalation-inhalation.

By adopting this assumption, we analyze primarily the impact of exhalation parameters on the amount of CO_2_ rebreathing appearing in the next inhalation. Air leakage, occurring mainly during inspiration, limits the volume of the inspiratory tidal volume but has a small impact on the exhalation parameters we analyze, which determine the amount of CO_2_ entering the distal part of the ventilatory circuit and affect CO_2_ rebreathing.

The air leak that may occur during exhalation has a lower value than during inhalation and, of course, may affect the amount of CO_2_ rebreathing, but in the same way as described above, i.e. reducing its amount.

Thus, air leaks appear to reduce CO_2_ rebreathing. Minimizing or even removing air leaks may improve noninvasive ventilation compliance but may intensify CO_2_ rebreathing during BIPAP ventilation.

## Conclusions

CO_2_ movement in the leak circuit during BIPAP ventilation was described and presented in a form of a scheme.

A close relationship was observed between the way of exhalation and the emergence of CO_2_ rebreathing. Based on CO_2_ movement 3 types of cycle were identified, type I and II do not induce rebreathing but type III does.

Using 16 ventilatory parameters (only the flow and pressure functions) measured or calculated for each of the 4747 tested respiratory cycles and a multilayer perceptron (MLP), a type of artificial neural network, it was possible to automatically classify the respiratory cycles into the three types with very high, 98.8% accuracy. This makes it possible to automatically detect on line CO_2_ rebreathing without the need to measure CO_2_ in the ventilator circuit. The system could be easy to implement as it uses pressure and flow signals commonly monitored in practically all modern ventilators.

The method described can allow to elaborate the systems of continuous warning the medical staff about the risk of CO_2_ rebreathing and perhaps, in future, a method of automatic modification of ventilatory parameters to reduce CO_2_ rebreathing and thus improve the patient's synchronization and compliance with the ventilator.

### Supplementary Information

Supplementary Information 1.

Supplementary Information 2.

Supplementary Information 3.

Supplementary Information 4.

Supplementary Information 5.

Supplementary Information 6.

## Data Availability

Raw data being the basis for the calculations made in the work are available in the Supplementary files section.
