# Supplementary material for: Automatic detection of CO2 rebreathing during BiPAP ventilation
Source: Sci Rep. 2024 Aug 17;14:19066. doi: 10.1038/s41598-024-63609-4 (PMC11330465; doi:10.1038/s41598-024-63609-4)
Supplement: Supplementary file 4 — Supplementary Information 4. [file 41598_2024_63609_MOESM4_ESM.docx]

**Detailed description of statistical methods assessing the classification of respiratory cycles into individual groups.**

As indicated in the main text, after the division of respiratory cycles into individual groups given in the Method, the ANOVA method was used.

It showed that all parameters differed significantly between the three types.

We don't know what caused the high ANOVA significance. These could be large differences between group means, but it could also be the fact that the individual cycles are not random samples from a given population, but are observations repeated on the same individuals. There were 18 subjects (patients), and the respiratory cycles received for them as many as n = 4747.

In short, the results from the ANOVA are indicative only: what if the assumptions for the ANOVA were met.

For this reason, for further analysis, we use Canonical Discriminant Analysis and machine learning methods commonly used in Pattern Recognition, which are very effective, and do not require the assumptions that ANOVA needs to say something.

The conclusion from the univariate ANOVA analysis is, that the values of the means of the 3 investigated groups are not identic. That entitles us to continue further the investigations in the direction of discriminant analysis using linear discriminant analysis and Fisher's principle to check the separation between the groups.

Visualization of the recorded data using Canonical Discriminant Analysis (CDA)

We have a data matrix containing n=4747 data vectors describing respiratory cycles. Each of them is characterized by *p=16* variables describing the given respiratory cycle. As described earlier the cycles were manually classified into 3 basic types denoted as type I, type II and type III appropriately. These values are referred to as true type value of the considered data vectors.

The CDA method constructs for this case of 3 groups of data to two (generally: number of groups minus one) new variables which maximize Fisher’s index of good discrimination (separation) between the groups of data viewed in the multivariate space. Fisher’s criterion is defined as the ratio of the between to the within group scatter. Fisher’s criterion can take values from the interval [0, +infinity], the larger the better. The CDA method computes for our 16-dimensional data vectors their two-dimensional representation {CD1, CD2} which may be displayed in the plane <CD1, CD2>. The representation obtained for our n=4747 data vectors is shown in Fig. 1.

Looking at that figure one may see there that points belonging to different groups are quite good separated. Fishers index for CV1 equals 9.2194, which means a good discriminative power. Looking that CV1 separates mainly type 3 from the other types. One may state also that the type 3 group the (blue squares) group is isolated quite clearly from the remaining two ones marked by magenta points (type 1) and the green points (denoting type 2).


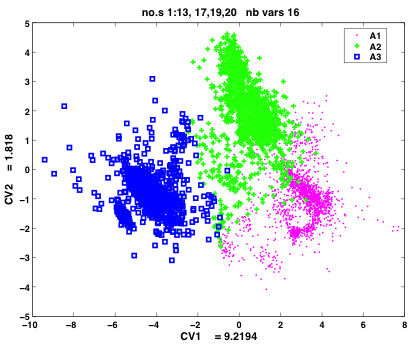


Fig 1. Discrimination analysis for 16 parameters: Pimax, iPPati, iPPate, Ti, Te, VtPati, VtPate, VtRespi, VtRespe, DebPatimx, DebPatemx, DebRespimx, DebRespemx, f, PEEP and NIV/INV . Lambda Wilk’s: 0.0347

Another index evaluating the discriminative power of some discriminant functions is the index called Wilk’s Lambda. It is defined as the ratio of the within group to the total scatter of the data and can take values from the interval [0, 1], the smaller, the better. For our data we have Wilk’s Lambda = 0.0347, which means a very good discriminative power.

The canonical discriminant functions CD1 and CD2 serving for evaluation of the coordinates displayed in Fig. 6 have as their basic goal a discriminative reduction of dimensionality of the analyzed data which are projected from the p-dimensional data space to the G-1 dimensional discriminant space. They do not aim at making a direct immediate classification of the projected data. To perform the classification (in our case to find for each of the respiratory cycles to which of the 3 types it belongs to, we have to use a surrogate method elaborated independently. To make the assignments to the proper type, we calculated the smallest Mahalanobis distance to the means of the respective 3 type groups. The obtained classification matrix is shown in Table 1.

| No of breaths in each group | % of breaths that were correctly classified | Breaths categorized by CDA | | |
| --- | --- | --- | --- | --- |
|  |  | Type I | Type II | Type III |
| I 1849 | 94.32 | 1744 | 0 | 105 |
| II 1545 | 90.1 | 117 | 1392 | 36 |
| III 1353 | 97.86 | 29 | 0 | 1324 |
| All 4747 | 93.95 | 1890 | 1392 | 1465 |

Table 1. Classification matrix using CDA model with 16 parameters. First column shows true counts n1, n2 and n3 of cycles classified by manually to types I, II, and III – as shown in the paragraph ‘a way to confirm the model’ above. Columns 3, 4, and 5 show the assignments to each group obtained when working with Canonical Discriminant functions yielding values shown in Fig. 1. Group assignments were done by calculating for each data vector the smallest Mahalanobis distance to the nearest type group center

The used method allows to correctly classify 94.32% of respiratory cycles to type I, 90.1% of cycles to type II and 97.86% of cycles to type III (Table 1, column 2).

The average correct classification rate is equal 93.95 %. Only about 6% of respiratory cycles were wrongly classified being assigned to the wrong group.
