# Supplementary material for: Automatic detection of CO2 rebreathing during BiPAP ventilation
Source: Sci Rep. 2024 Aug 17;14:19066. doi: 10.1038/s41598-024-63609-4 (PMC11330465; doi:10.1038/s41598-024-63609-4)
Supplement: Supplementary file 5 — Supplementary Information 5. [file 41598_2024_63609_MOESM5_ESM.docx]

Figure of MLP network proposed in the study and a table of weight estimates.

Fig . Figure of MLP network proposed in this study.

| **Parameter Estimates** | | | | | | | | | | | | | | | | | | |
| --- | --- | --- | --- | --- | --- | --- | --- | --- | --- | --- | --- | --- | --- | --- | --- | --- | --- | --- |
| Predictor | | Predicted | | | | | | | | | | | | | | | | |
|  |  | Hidden Layer 1 | | | | | | | | Hidden Layer 2 | | | | | | Output Layer | | |
|  |  | H(1:1) | H(1:2) | H(1:3) | H(1:4) | H(1:5) | H(1:6) | H(1:7) | H(1:8) | H(2:1) | H(2:2) | H(2:3) | H(2:4) | H(2:5) | H(2:6) | [type=1] | [type=2] | [type=3] |
| Input Layer | (Bias) | -,718 | ,440 | -,429 | -1,307 | -,003 | ,752 | 2,138 | -2,296 |  |  |  |  |  |  |  |  |  |
|  | InspmaximalpressurePatsidecmH2O | -1,279 | 2,622 | ,426 | -,300 | 1,205 | 1,170 | ,082 | -,390 |  |  |  |  |  |  |  |  |  |
|  | Insptimes | -,004 | -,037 | -,093 | ,178 | -,938 | ,085 | -,019 | ,822 |  |  |  |  |  |  |  |  |  |
|  | Exptimes | -1,362 | ,783 | ,453 | -,378 | -,766 | ,046 | ,231 | -,753 |  |  |  |  |  |  |  |  |  |
|  | InspsurfaceunderpressurecurvePatsidecm2 | -,708 | 1,566 | ,585 | -,399 | ,680 | 1,034 | -,581 | ,344 |  |  |  |  |  |  |  |  |  |
|  | ExpsurfaceunderpressurecurvePatsidecm2 | -,591 | 1,276 | ,588 | 1,058 | ,162 | 1,383 | ,355 | -,031 |  |  |  |  |  |  |  |  |  |
|  | InsptidalvolumePatsideml | -,150 | -,880 | -1,306 | -,760 | -,565 | -,064 | 1,146 | -,505 |  |  |  |  |  |  |  |  |  |
|  | ExptidalvolumePatsideml | -,887 | -1,190 | -1,219 | -1,397 | -,492 | -,540 | 2,448 | -2,409 |  |  |  |  |  |  |  |  |  |
|  | InsptidalvolumeVentsideml | -,573 | ,190 | -,222 | ,194 | -,358 | ,042 | ,154 | -,450 |  |  |  |  |  |  |  |  |  |
|  | ExptidalvolumeVentsideml | ,269 | -2,149 | -2,393 | -2,252 | -,779 | -1,796 | 2,502 | -2,773 |  |  |  |  |  |  |  |  |  |
|  | InspmaximalflowPatsidemls | -,944 | ,613 | -,417 | ,342 | -1,122 | ,252 | -,573 | ,605 |  |  |  |  |  |  |  |  |  |
|  | Expiratorymaximalflowpatsidemls | -,894 | -,440 | -,098 | -,407 | -,427 | -,072 | -,568 | -,797 |  |  |  |  |  |  |  |  |  |
|  | InspiratorymaximalflowVentsidemls | -,672 | ,661 | ,266 | ,186 | ,155 | ,997 | ,608 | ,671 |  |  |  |  |  |  |  |  |  |
|  | ExpiratorymaximalflowVentsidemls | -,487 | -,680 | -,563 | -1,623 | -,459 | -,245 | ,863 | -2,174 |  |  |  |  |  |  |  |  |  |
|  | InvasiveNonInvasive | -,861 | ,751 | ,693 | 1,047 | ,448 | 1,576 | ,775 | -,334 |  |  |  |  |  |  |  |  |  |
|  | Frequencyofbreathingperminutemin | ,675 | ,034 | -,979 | -,568 | ,426 | ,288 | ,866 | 1,164 |  |  |  |  |  |  |  |  |  |
|  | PositiveendexpiratorypressurecmH2O | -,743 | 3,040 | ,097 | ,576 | ,910 | 2,400 | -,308 | ,632 |  |  |  |  |  |  |  |  |  |
| Hidden Layer 1 | (Bias) |  |  |  |  |  |  |  |  | ,659 | -1,391 | -,783 | ,407 | 1,572 | -,410 |  |  |  |
|  | H(1:1) |  |  |  |  |  |  |  |  | ,566 | ,305 | -,164 | ,979 | ,086 | -,500 |  |  |  |
|  | H(1:2) |  |  |  |  |  |  |  |  | -1,794 | ,082 | -,169 | -2,054 | -,180 | 1,507 |  |  |  |
|  | H(1:3) |  |  |  |  |  |  |  |  | -3,066 | ,793 | ,193 | -3,634 | -,881 | 2,308 |  |  |  |
|  | H(1:4) |  |  |  |  |  |  |  |  | -,142 | 2,182 | 1,729 | ,225 | -1,060 | -,059 |  |  |  |
|  | H(1:5) |  |  |  |  |  |  |  |  | -1,206 | -1,398 | -,009 | -,661 | ,789 | -,560 |  |  |  |
|  | H(1:6) |  |  |  |  |  |  |  |  | -1,971 | ,573 | ,104 | -2,779 | -,768 | 2,572 |  |  |  |
|  | H(1:7) |  |  |  |  |  |  |  |  | 1,265 | -2,855 | -1,666 | 1,908 | 3,780 | -1,592 |  |  |  |
|  | H(1:8) |  |  |  |  |  |  |  |  | ,037 | 2,300 | 1,519 | -,505 | -2,169 | 1,269 |  |  |  |
| Hidden Layer 2 | (Bias) |  |  |  |  |  |  |  |  |  |  |  |  |  |  | -1,019 | 1,750 | -,731 |
|  | H(2:1) |  |  |  |  |  |  |  |  |  |  |  |  |  |  | -1,310 | -2,526 | 3,331 |
|  | H(2:2) |  |  |  |  |  |  |  |  |  |  |  |  |  |  | 4,230 | -2,531 | -1,256 |
|  | H(2:3) |  |  |  |  |  |  |  |  |  |  |  |  |  |  | 2,699 | -1,930 | -1,022 |
|  | H(2:4) |  |  |  |  |  |  |  |  |  |  |  |  |  |  | -2,990 | -2,520 | 5,353 |
|  | H(2:5) |  |  |  |  |  |  |  |  |  |  |  |  |  |  | -4,443 | 3,205 | 1,413 |
|  | H(2:6) |  |  |  |  |  |  |  |  |  |  |  |  |  |  | 2,705 | 1,129 | -3,179 |

Table. Table of weight estimates.
