# Supplementary material for: Automatic detection of CO2 rebreathing during BiPAP ventilation
Source: Sci Rep. 2024 Aug 17;14:19066. doi: 10.1038/s41598-024-63609-4 (PMC11330465; doi:10.1038/s41598-024-63609-4)
Supplement: Supplementary file 6 — Supplementary Information 6. [file 41598_2024_63609_MOESM6_ESM.docx]

Patients characteristics.

| **Table 1 Patients’ characteristics** | | | | |
| --- | --- | --- | --- | --- |
| **Patient** | **Sex** | **Age** | **VD** | **Diagnosis** |
| 1 | F | 45 | 21 | Bone marrow transplant, uro-sepsis |
| 2 | M | 60 | 16 | Status epilepticus, pneumonia |
| 3 | M | 61 | 2 | Suicide attempt |
| 4 | F | 30 | 1 | Thoracic trauma |
| 5 | F | 25 | 2 | Suicide attempt |
| 6 | M | 37 | 2 | Suicide attempt, pneumonia |
| 7 | M | 76 | 7 | Pneumonia, cardiac arrest |
| 8 | M | 33 | 3 | Abdominal surgery |
| 9 | M | 67 | 14 | Aspiration pneumonia |
| 10 | M | 58 | 5 | Left cardiac insufficiency, COPD |
| 11 | M | 26 | 2 | Mediastinal surgery, cystic fibrosis |
| 12 | M | 50 | 17 | Abdominal surgery, sepsis, ARDS, emphysema |
| 13 | M | 60 | 10 | Pneumonia on COPD |
| 14 | M | 90 | 3 | COPD exacerbation |
| 15 | M | 66 | 16 | Pneumonia, septic shock |
| 16 | F | 55 | 14 | Pulmonary embolism, sepsis |
| 17 | M | 57 | 10 | Duodenal hemorrhage, sepsis |
| *18* | F | 82 | 3 | COPD exacerbation |
| VD, ventilation duration | | | | |
